# Interplay between pro-inflammatory cytokines and growth factors in depressive illnesses

**DOI:** 10.3389/fncel.2013.00068

**Published:** 2013-05-10

**Authors:** Marie-Claude Audet, Hymie Anisman

**Affiliations:** Department of Neuroscience, Carleton UniversityOttawa, ON, Canada

**Keywords:** antidepressant, BDNF, depression, growth factors, pro-inflammatory cytokines, stressors

## Abstract

The development of depressive disorders had long been attributed to monoamine variations, and pharmacological treatment strategies likewise focused on methods of altering monoamine availability. However, the limited success achieved by treatments that altered these processes spurred the search for alternative mechanisms and treatments. Here we provide a brief overview concerning a possible role for pro-inflammatory cytokines and growth factors in major depression, as well as the possibility of targeting these factors in treating this disorder. The data suggest that focusing on one or another cytokine or growth factor might be counterproductive, especially as these factors may act sequentially or in parallel in affecting depressive disorders. It is also suggested that cytokines and growth factors might be useful biomarkers for individualized treatments of depressive illnesses.

Major depression is not only the most common neuropsychiatric disorder, but it is also highly comorbid with other pathological conditions, including anxiety disorders, schizophrenia, heart diseases, auto-immune illnesses, metabolic syndrome, diabetes, neurodegenerative disorders, and post-stroke neuropsychiatric complications (Anisman et al., 2008). Unfortunately, the available treatment strategies have not been overwhelmingly successful in attenuating depressive symptoms in all patients, and the specific processes involved in the pathogenesis of depressive illnesses have yet to be fully identified. In this regard, in the past decade there has been increasing attention devoted to the possibility that both inflammatory and growth factors might play a provocative role in depression. In the present report we will explore the possibility that pro-inflammatory cytokines, which had traditionally been thought of as signaling molecules of the inflammatory immune system, but which are also endogenously produced in the brain, might come to interact with growth factors, thereby influencing neuronal and synaptic growth and plasticity, and ultimately promoting depressive illnesses.

## Implications of the inflammatory immune system in depressive illnesses

The search for new antidepressant strategies has recently generated a growing interest especially as a significant percentage of depressed patients do not respond positively to medication. In fact, traditional treatments aimed at increasing monoamine concentrations, including tricyclic antidepressants (TCAs), monoamine oxidase inhibitors (MAOIs), selective serotonin reuptake inhibitors (SSRIs), or serotonin-norepinephrine reuptake inhibitors (SNRIs), attenuate depressive symptoms in only 50–60% of individuals, although combination drug therapies can yield appreciably improved success rates (Blier et al., 2010). When drug treatments are effective in reducing depression, remission is often incomplete and the relapse rates may exceed 50% over a 5-year period (Moncrieff and Kirsch, 2005). As antidepressant effects are usually observed only after 2–3 weeks of treatment, patients may also be at risk for harm during this period, especially as multiple attempts might be needed before an effective treatment is found. As well, several undesirable side effects frequently accompanied antidepressant therapy, including agitation, sexual dysfunction, and weight gain, which might precipitate treatment discontinuation or patients taking “drug holidays.” Thus, identification of additional processes involved in depression has been the focus of intensive efforts in order to develop alternative treatments or multi-targeted therapies aimed at attenuating symptoms sooner and more efficiently.

### Increased activity of pro-inflammatory cytokines in individuals with major depression and in animal models of the disorder

One research avenue that has received considerable attention has concerned the possibility that increased activity of pro-inflammatory cytokines might contribute to the pathogenesis of depressive illnesses (Maes, 1995). Pro-inflammatory cytokines are signaling molecules of the inflammatory immune system that initiate and coordinate the cascade of immune events necessary to deal with infection, toxins, injury, and/or trauma. Table 1 shows a subset of the many cytokines that have been identified in circulation as well as in various brain regions, including those that have anti-inflammatory properties. Importantly, these cytokines have been hypothesized as being involved in the provocation of mood disorders, such as depressive illnesses.

**Table 1:** **Subset of pro- and anti-inflammatory cytokines that had been implicated in depressive illnesses: potential roles in behavioral processes and/or neuropsychiatric disorders**.

	**Cytokine name**	**Immune cells (main source)**	**Major actions**	**Major outcomes**	**Behavioral processes/ neuropsychiatric disorders**
**PRO-INFLAMMATORY**					
Interleukins	IL-1β	Macrophages, monocytes, dendritic cells	Stimulates immune cells and pro-inflammatory cytokines, activates microglia, regulates growth factor activity	Acute phase response, fever, wound healing, pain hypersensitivity, angiogenesis	Sickness behaviors, stress response, cognitive processes, depression, anxiety, schizophrenia, Alzheimer's disease
	IL-2	Th1 cells	Growth and differentiation of T cells	Immune homeostasis	Depression, schizophrenia
	IL-6	Macrophages, Th2 cells	Synthesis of acute phase proteins, growth and differentiation of T and B cells, secretion of antibodies. Regulates pro-inflammatory factors (anti-inflammatory actions)	Acute phase response, fever, fighting infection	Stress response, depression, post-traumatic stress disorder (PTSD), Alzheimer's disease, schizophrenia
	IL-18	Macrophages, dendritic cells	Stimulates maturation of T and NK cells, stimulates production of IFN-γ, negatively regulates IL-4	Regulates homeostasis	Post-stroke depression, hyperphagia, metabolic syndrome
Interferons	IFN-α	B, T, and NK cells	Activates macrophages and NK cells	Flu-like symptoms (e.g., fever)	Depression, cognitive processes (delirium)
	IFN-γ	Th1 and NK cells	Activates macrophages and NK cells, activates microglia	Flu-like symptoms (e.g., fever), anti-tumoral	Emotionality disturbances
Tumor necrosis factor	TNF-α	Macrophages, Th1 cells, NK cells, mastocytes	Stimulates immune cells, activates microglia, systemic inflammation, tissue destruction	Acute phase response, fever, sepsis	Sickness behaviors, depression, autoimmune diseases
Other factors	MIF	Macrophages, fibroblasts	Blocks anti-inflammatory effects of glucocorticoids	Enhances inflammatory response, neurogenesis	Depression
**ANTI-INFLAMMATORY**					
Interleukins	IL-4	Th2 cells, mastocytes, basophils	Stimulates differentiation of Th2 and B cells, decreases production of Th1 cells	Limits pathological inflammation	Allergies, ischemic stroke, auto-immune diseases, suicide
	IL-10	Monocytes, Th1 and Th2 cells	Decreases pro-inflammatory cytokines from macrophages and Th1 cells, stimulates Th2 and B cells, inhibits NK cells	Represses inflammatory immune response	Depression, schizophrenia

Consistent with a role for cytokines in depression, meta-analyses have established that circulating concentrations of the pro-inflammatory cytokines interleukin (IL)-6 and tumor necrosis factor (TNF)-α were elevated in non-medicated depressed patients, whereas variations of IL-1β were less consistently demonstrated (Dowlati et al., 2010; Liu et al., 2012a), possibly because of the difficulty in detecting the very low levels of this interleukin in human circulation. In contrast, lower serum levels of the anti-inflammatory cytokine IL-10 and higher IL-6/IL-10 ratios were found in drug-free depressed individuals, and low IL-10 levels were negatively correlated to depressive scores (Dhabhar et al., 2009). In keeping with the view that stressful events might influence cytokine levels and thus promote depression, a meta-analysis revealed that psychosocial stressors increased plasma concentrations of pro-inflammatory cytokines, especially that of IL-6 (Steptoe et al., 2007), and this outcome was greater among individuals with a history of childhood maltreatment (Carpenter et al., 2010), a condition that also favors the development of depression. Plasma IL-1β concentrations were also elevated after psychosocial stressors in humans, but this change was fairly transient (Steptoe et al., 2007; Yamakawa et al., 2009), again supporting the view that this cytokine in the blood might play a less prominent role in depression, although these data may not speak to the central involvement of IL-1β in mediating this disorder.

Parenthetically, even though the available data point to the fundamental involvement of IL-6 in relation to depressive illnesses, this should not be taken to mean that other cytokine variations, especially those that occur in the brain, are not relevant to the evolution of depression. In fact, a small portion of the pro-inflammatory cytokines released into circulation can gain access to the brain at the blood-brain barrier and spread through volume diffusion at circumventricular sites (Vitkovic et al., 2000) or may reach the brain through saturable transport systems (Banks, 2006). In addition, brain microglia may produce pro-inflammatory cytokines in response to inflammatory stimuli or stressors (Quan et al., 1998; Dantzer et al., 2008; Sukoff Rizzo et al., 2012). The elevations of pro-inflammatory cytokines are not restricted to regions that are associated with hormonal changes (e.g., hypothalamus), but have been documented in the prefrontal cortex (PFC), hippocampus, amygdala, as well as other brain regions that are involved in stressor appraisal processes and depressive illnesses (Anisman et al., 2008).

Among the few studies that have examined brain cytokine variations associated with depressive illnesses, several pro-inflammatory cytokines, including IL-1 and a precursor of the soluble form of TNF-α, TNF trans-membrane, were reported to be up-regulated in post-mortem PFC of patients with major depression (Dean et al., 2010; Shelton et al., 2011). Increased IL-1β, IL-6, and TNF-α in post-mortem PFC were also found in teenaged individuals that died by suicide, although in this study not all tissue had come from individuals that had been diagnosed with major depression (Pandey et al., 2012). A potential role for brain cytokine elevations in depression has been confirmed in studies using rodent models of the disorder showing that psychosocial stressors, such as social defeat, increased plasma IL-6, and IL-1β levels and up-regulated expression of these cytokines in the PFC and hippocampus (Audet et al., 2010, 2011). Likewise, fairly strong stressors such as tailshock, footshock, and immobilization increased IL-1β protein and mRNA in serum and in hypothalamus (O'Connor et al., 2003; Deak et al., 2005) as well as IL-6 in the frontal cortex (Sukoff Rizzo et al., 2012). Interestingly, cortical elevations of IL-6 promoted by inescapable footshock were apparent only in rats that had developed a depressive phenotype, suggesting that IL-6 activity (at least in the frontal cortex) might be particularly aligned with the emergence of depressive features secondary to stressor exposure (Sukoff Rizzo et al., 2012).

The implications of pro-inflammatory cytokine activation in relation to depressive illnesses appear to be particularly relevant to depression that is not readily abated by monoamine-based antidepressants (Maes et al., 2009). It has, in fact, been suggested that depressed patients who repeatedly fail to respond to traditional antidepressants may exhibit a distinctive pro-inflammatory profile (Krishnadas and Cavanagh, 2012). Consistent with this perspective, up-regulated IL-1β and TNF-α serum mRNA expression prior to treatment was more pronounced among depressed patients who did not respond to later SSRIs or TCAs compared to those who did, and was negatively correlated with clinical outcomes (Cattaneo et al., 2013). In this particular report, basal cytokine expression predicted antidepressant efficacy and was reduced by treatment, but only IL-6 down-regulation was specific to treatment responders (Cattaneo et al., 2013). Likewise, the elevated circulating levels of IL-6 prior to medication was more pronounced in depressed patients that did not respond to later treatment with TCAs, SSRIs, or SNRIs compared to those who did respond, and IL-6 elevations were attenuated only in those patients who responded positively to antidepressants (Lanquillon et al., 2000; O'Brien et al., 2007; Yoshimura et al., 2009). The possibility that pre-existing activation of inflammatory processes might promote resistance to antidepressants has also been confirmed in animal studies. For instance, the positive actions of fluoxetine were precluded if chronically stressed rats had been treated with the bacterial endotoxin lipopolysaccharide (LPS) prior to each stressor treatment (Wang et al., 2011a). A blunted response to the antidepressant effects of fluoxetine was also observed in mice engineered to overexpress IL-6 in the frontal cortex and hippocampus as well as in mice that had received i.c.v. injections of IL-6, suggesting that brain elevations of this cytokine might be particularly important contributors to antidepressant resistance (Sukoff Rizzo et al., 2012).

There is some question concerning the clinical significance of cytokine changes in periphery and in the brain in relation to depression. In fact, as informative as peripheral cytokine activity might be, depressive illnesses ultimately are likely more closely aligned with brain cytokine variations (either endogenously produced or peripherally stimulated) or with effects secondary to such changes, including variations of neurotransmitters, hormones, or growth factors. What circulating variations of cytokines in depressed individuals might reflect in relation to the actual disorder is still uncertain, especially as clinical improvements elicited by antidepressants are not uniformly accompanied by normalization of peripheral cytokine levels (Eller et al., 2009; Hannestad et al., 2011). Circulating cytokines might be peripheral biomarkers or trait characteristics of depressive illnesses, as they might also reflect distress experienced by depressed individuals. It is also possible that blood cytokine variations may be indicative of those individuals who are at risk for depression or they may be suggestive of an ongoing biological dysfunction that signifies which individuals will be more resistant to antidepressants or particularly prone to relapse following successful therapy. In fact, the specific contributions of circulating versus brain pro-inflammatory cytokines in mediating depressive symptoms remain to be addressed and whether peripheral cytokine changes might predict brain variations that are translated into depressive conditions is uncertain.

To this point, we focused primarily on the influence of pro-inflammatory cytokines in relation to behavioral disturbances. However, this should not be misconstrued to suggest that peripheral factors do not play a role in this regard. For instance, peripheral epinephrine may stimulate immune activity and increase cytokine release from immune cells, whereas circulating cortisol/corticosterone may have the opposite effects (Anisman et al., 2008). Once released, as described earlier, these cytokine changes can directly or indirectly influence central neurochemical functioning. The use of β-adrenergic receptor antagonists as adjunctive treatment was shown to accelerate and accentuate clinical improvements elicited by SSRIs in patients with major depression (Portella et al., 2011), or in those that experienced depressive symptoms post-surgery (Battes et al., 2012). Moreover, in mice, pretreatment with the β-adrenergic antagonist propranolol reversed anxiety-like behaviors and normalized stressor-induced plasma and brain IL-6 and TNF-α reactivity elicited by a social stressor (Wohleb et al., 2011; Hanke et al., 2012), thus suggesting a potential role for epinephrine in cytokine-mediated mood disturbances elicited by stressors.

In addition to the involvement of epinephrine in immunity and mood disorders, it has been known for some time that increased cholinergic functioning can affect immune cell activity and influence depressive-like states (Blalock, 1994; Ofek et al., 2007; Pavlov et al., 2009; Ofek and Soreq, 2013). In this regard, cholinesterase inhibitors, that may act to block peripheral inflammatory processes, attenuated comorbid depressive symptoms in patients with Alzheimer's disease (Spalletta et al., 2012). However, a meta-analysis revealed that the use of cholinesterase inhibitors as adjunctive antidepressant therapy had no clear benefit in older adults with depression (McDermott and Gray, 2012), suggesting that blood cytokine reductions brought about by acetylcholine manipulations might not be sufficient to attenuate depressive symptoms in patients with major depressive disorder.

### Depressive episodes associated with immunotherapy or inflammatory conditions

Non-medicated patients suffering from chronic inflammatory illnesses (e.g., multiple sclerosis, chronic hepatitis C, cancer) or acute inflammatory conditions (e.g., stroke, surgery) commonly experienced comorbid depressive symptoms (Musselman et al., 2001b; Cremeans-Smith et al., 2009). Such findings, of course, are correlational and hence do not provide a causal link between inflammatory factors and depressive states. However, strong evidence causally linking pro-inflammatory cytokines to depression comes from many reports showing that immunotherapy with interferon (IFN)-α in the treatment of hepatitis C or some types of cancer promoted a syndrome similar to that of major depression, sometimes sufficiently severe to necessitate treatment discontinuation (Capuron and Miller, 2004). Although the depressive symptoms generally resolved when IFN-α therapy ceased, patients were often sensitized so that they were particularly reactive to further cytokine challenges (e.g., subsequent treatment) (Loftis and Hauser, 2004).

The development of depression among individuals receiving IFN-α immunotherapy may be influenced by many of the same factors that predict major depression under other conditions. In particular, subsyndromal levels of depression prior to immunotherapy predicted more intense depressive episodes in response to the IFN-α treatment (Capuron et al., 2004; Beratis et al., 2005), and depressive symptoms stemming from the cytokine therapy were more prominent among individuals with poor social support resources (Capuron et al., 2004). Furthermore, low levels of tryptophan (Capuron et al., 2003a) as well as elevated levels of adrenocorticotropic hormone and cortisol (Capuron et al., 2003b) in response to IFN-α were predictive of depressive illnesses. Paralleling the effects of manipulations that reduced tryptophan levels (Young and Leyton, 2002; Neumeister et al., 2004), depressive symptoms engendered by IFN-α were more pronounced among women and among those with a history of depression (Capuron et al., 2003a). Finally, as expected based on a cytokine perspective of the disorder, depression during IFN-α treatment was most likely to be instigated among those individuals with the highest baseline levels of soluble IL-2 receptor (sIL-2r), IL-6, and IL-10 (Wichers et al., 2006). It thus seems that the very same factors that enhance vulnerability to stressor-related depression also increase the occurrence of depressive symptoms in response to IFN-α immunotherapy.

Despite the limited success that has been reported with antidepressants in the treatment of depression instigated by life stressors or related factors, SSRIs, such as sertraline, paroxetine, and citalopram, were reported to be effective in diminishing depressive symptoms otherwise produced by IFN-α (Musselman et al., 2001a; Hauser et al., 2002; Maddock et al., 2004; Baraldi et al., 2012). In this regard, paroxetine primarily affected the mood-related symptoms elicited by IFN-α, but had only modest effects on fatigue and anorexia (Musselman et al., 2001a; Raison et al., 2005). These findings are of theoretical significance in linking IFN-α immunotherapy to depression, but also have practical, clinical relevance as antidepressants may improve IFN-α tolerability, thus enhancing treatment continuation and effectiveness.

It has to be taken into account that emotional distress associated with experiencing chronic and/or severe pathological conditions such as hepatitis C, cancer, or stroke may contribute to the development of depression (Anisman, 2009). As indicated earlier, stressors ordinarily promote cytokine variations in plasma as well as in brain areas known to be involved in depression (Steptoe et al., 2007; Audet et al., 2010) and thus, could potentially influence consequences associated with treatments such as IFN-α. In fact, immunogenic treatments (e.g., bacterial endotoxin) applied on a stressor backdrop may synergistically influence these stress-induced cytokine outcomes, as reported with respect to monoamines turnover (Gibb et al., 2011). Thus, it is possible that the enhanced inflammation associated with immune-related conditions or that elicited by IFN-α immunotherapy, when coupled with the effects associated with the distress of illness, may promote cytokine and other biological changes (e.g., monoamine turnover) that exceed those elicited by either condition alone, thereby increasing the likelihood of depression emerging. In effect, the consequences of inflammatory changes on depressive disorders might reflect the conjoint actions of these cytokines and the stressor backdrop upon which they appear.

### Potential processes involved in pro-inflammatory cytokine provocation of depression

The specific processes by which activation of the inflammatory immune system might come to trigger or exacerbate depression have not been fully identified, although several candidates are available in this regard. It has been proposed that activation of cytokines in the brain (elicited by peripheral inflammation, IFN-α immunotherapy, tissue damage, or stressors/distress) might stimulate the enzyme indoleamine 2,3-dioxygenase (IDO), which is responsible for the degradation of tryptophan within the kynurenine pathway (O'Connor et al., 2009; Zunszain et al., 2012). Acceleration of tryptophan catabolism would limit its availability and thus result in diminished serotonin (5-HT) synthesis, which might favor the development of depressive illnesses (Dantzer et al., 2011; Maes et al., 2011). Alternatively, the increased rate of tryptophan degradation caused by IDO activation may enhance the production of *N*-methyl-D aspartate (NMDA) receptor antagonists (e.g., kynurenic acid) or agonists (e.g., quinolinic acid) as well as other neurotoxins (e.g., 3-hydroxykynurenine). These variations within the kynurenine pathway would then cause the activation of oxidative effects, elicit mitochondrial dysfunctions, and engender neurotoxic effects that culminate in depression (Dantzer et al., 2011; Maes et al., 2011).

It is likely, however, that brain cytokines influence processes beyond those involving 5-HT or those stemming from neurotoxic changes subsequent to IDO activation. In this regard, it has been suggested that stimulation of brain inflammatory processes might alter neurogenesis and neuroplasticity, known to play an important role in depression, through cytokine actions on different families of growth factors implicated in the growth, differentiation, vascularization, and survival of neurons and synapses (Duman and Monteggia, 2006; Miller et al., 2009). Specifically, brain pro-inflammatory cytokines might interact with neurotrophins, including brain-derived neurotrophic factor (BDNF) and nerve growth factor (NGF), fibroblast growth factors, including basic fibroblast growth factor (FGF-2), as well as vascular endothelial growth factor (VEGF), and erythropoietin (EPO), and favor the emergence of depressive features (see Anisman and Hayley, 2012). Alternatively, it is possible that activation of growth factors (e.g., through the administration of antidepressants, physical exercise, or environmental enrichment) or even direct administration of growth factors (as an antidepressant strategy) may come to attenuate depressive symptoms by influencing brain cytokine activity. In the next sections, the importance of adult neurogenesis and growth factors, especially BDNF, in relation to depression will be described and the potential interplay between pro-inflammatory cytokines and these factors in promoting depressive illnesses will be discussed.

## Neurogenesis, growth factors, and depression: a focus on BDNF

The generation of new neurons from neural stem cells located within the subgranular zone of the dentate gyrus of the hippocampus (SGZ) and the subventricular zone of the lateral ventricle (SVZ) and their functional integration to mature neural networks may occur in the adult brain. This process, adult neurogenesis, has received increasing attention owing to its important role in neuronal and synaptic plasticity and in promoting particular behavioral outcomes. As shown in Table 2, several growth factors may contribute significantly to adult neurogenesis by promoting the growth, differentiation, maintenance, and survival of new neurons and synapses (e.g., in the case of neurotrophins such as BDNF or NGF, or of FGFs), or by stimulating the growth of new blood vessels required for vascularization of brain tissue (e.g., in the case of VEGF or FGF-2). Because of their sensitivity to environmental stimuli and their high degree of plasticity, newborn neurons may contribute to stress responsiveness and adaptation as well as learning and memory (Castilla-Ortega et al., 2011). Accordingly, abnormalities in adult neurogenesis as well as impairments of growth factors functioning, especially in the hippocampus, have been associated with the emergence of neuroaffective and neurocognitive symptoms, including those related to depressive illnesses (Kempermann and Kronenberg, 2003; Pittenger and Duman, 2008).

**Table 2:** **Subset of growth factors that had been implicated in depressive illnesses: potential roles in behavioral processes and/or neuropsychiatric disorders**.

**Growth factor family**	**Growth factor name**	**Major outcomes**	**Behavioral processes/neuropsychiatric disorderss**
Neurotrophins	BDNF	Growth and differentiation of neurons, survival of existing neurons and synapses, neuronal and synaptic plasticity	Response and adaptation to stress, learning and memory, social behaviors. Depression, anxiety.
	NGF	Growth and differentiation of neurons, maintenance and survival of existing neurons, neuronal and synaptic plasticity	Response to stress, learning and memory. Depression, anxiety. Antidepressant effects.
Fibroblast growth factors	FGF-2	Development of nervous system, differentiation of stem cells, wound healing, angiogenesis	Response to stress, cognitive processes, social behaviors. Depression, schizophrenia. Antidepressant effects.
Vascular endothelial growth factors	VEGF	Angiogenesis, vasculogenesis	Social behaviors. Depression, schizophrenia, anxiety. Antidepressant effects.
	EPO	Production of blood cells (erythropoiesis), increases oxygen delivery, wound healing, angiogenesis, neuronal protection	Cognitive performance. Antidepressant effects.

### Impairments in adult neurogenesis in relation to depressive illnesses

Smaller hippocampal volumes, thought to reflect reduced neurogenesis and/or neuron survival, have been reported in patients with first episode depression as well as in those with recurrent depression (Sheline et al., 1996; Frodl et al., 2002; MacQueen et al., 2003; Cole et al., 2011). Post-mortem brain analyses among non-medicated depressed patients and controls, however, have not fully succeeded in detecting differences in the number and proliferation of neural stem cells in the dentate gyrus (Reif et al., 2006; Boldrini et al., 2009) or disturbances of the cytoarchitecture of the SVZ (Comte et al., 2012). This said, it was observed that the expression of two neuroplastic markers, doublecortin and polysialylated-neural cell adhesion molecule, was elevated in the basolateral amygdala of depressed individuals (Maheu et al., 2013). This finding is inconsistent with common views concerning the relationship between impairments of neurogenesis and depression, but as the increased expression of these markers was apparent in the amygdala, it is possible that this had reflected anxiety or elevated stressor reactivity that is frequently comorbid with depressive disorders.

A role for adult neurogenesis in depression has come principally from the observation that antidepressants promoted neuronal growth and plasticity or attenuated impairments of neurogenesis. In this regard, more neural stem cells and capillaries were found in post-mortem dentate gyrus of depressed patients treated with SSRIs compared to non-medicated depressed patients or healthy controls (Boldrini et al., 2009, 2012), a finding that was also observed in rats wherein chronic SSRI administration promoted neuron proliferation in the hippocampus and the PFC (Malberg et al., 2000; Kodama et al., 2004). Importantly, among repeatedly stressed non-human primates, enhanced hippocampal neurogenesis during SSRI treatment was necessary for clinical improvements to occur (Perera et al., 2011). This said, there have also been reports that antidepressants had no effects on hippocampal neurogenesis (Cowen et al., 2008), and even reduced neurogenesis in the SVZ (Ohira and Miyakawa, 2011).

### BDNF variations in depressed patients

Much of the data supporting a role for impairments of adult neurogenesis in the emergence of depression has come from the observation that the neurotrophin BDFN, which plays a fundamental role in neurogenesis and neuron survival, was deregulated in depressed patients or as a result of stressor experiences (e.g., depression secondary to stressful events). In line with this perspective, reduced serum levels and mRNA expression of BDNF were reported in drug-free depressed patients compared to those treated with antidepressants or healthy controls (Karege et al., 2002; Shimizu et al., 2003; Molendijk et al., 2011; Cattaneo et al., 2013), and low BDNF levels were negatively correlated with symptom severity (Shimizu et al., 2003) although it has also been found that BDNF levels did not parallel core clinical features of depression, including severity of symptoms (Molendijk et al., 2011). Interestingly, reduced hippocampal volume in drug-free patients suffering from first-episode major depression was more pronounced among those with lower serum BDNF concentrations (Eker et al., 2010). This outcome was also observed among individuals carrying the BDNF Val^66^Met polymorphism (Molendijk et al., 2012), a genetic mutation in which valine (Val) is replaced by methionine (Met) on one or both alleles of the gene for BDNF, resulting in diminished secretion of the neurotrophin, and thus impaired neurogenesis.

Direct evidence of altered BDNF in the human depressed brain appears to be limited to post-mortem analyses showing reduced protein density of pro-BDNF in hippocampus (Dunham et al., 2009), down-regulated mRNA expression of the BDNF receptor TrkB in the subgenual anterior cingular cortex (Tripp et al., 2012), and reduced pro- and mature BDNF protein and mRNA in amygdala (Guilloux et al., 2012). As well, lower BDNF mRNA expression in post-mortem PFC and hippocampus were reported individuals that had died through suicide (Dwivedi et al., 2003).

With respect to the possibility that antidepressants might stimulate BDNF activity (and thus neurogenesis), post-mortem analyses indicated that hippocampal concentrations of the neurotrophin were elevated among depressed individuals who had been treated with antidepressants compared to those who had not been medicated (Chen et al., 2001). Circulating elevations of BDNF elicited by antidepressants were also positively correlated with clinical improvements and in some cases occurred only in those patients that responded to treatment (Lee and Kim, 2008; Yoshimura et al., 2009; Cattaneo et al., 2013), supporting the possibility that attenuation of depressive symptoms elicited by antidepressants were attributable to BDNF normalization. When considered together with clinical progress, it was shown that early plasma BDNF increases (e.g., 7 days after the beginning of treatment) predicted successful responses to antidepressant treatment (Dreimüller et al., 2012). These data are, of course, only correlational and it seems that symptom improvements following antidepressants might not consistently be accompanied by BDNF normalization. As a matter of fact, although they were equally effective in improving depressive symptoms, the SNRI venlafaxine, the SSRI sertraline, and the TCA amitriptyline increased BDNF levels, whereas neurotrophin concentrations remained unchanged after treatment with the SSRI escitalopram and even declined after the SSRI paroxetine (Hellweg et al., 2008; Matrisciano et al., 2009). Whereas these findings could be fortuitous, they might also suggest selectivity regarding the effects of particular antidepressants on the neurotrophin, or simply indicate that additional processes are involved in the clinical effects of antidepressants.

### Stressor effects on BDNF: potential moderating factors

As briefly mentioned earlier, additional support for the possibility that impairments of neurogenesis may promote depression has come from a series of reports indicating that stressors elicit peripheral and central variations of growth factors, especially that of BDNF. For example, in humans, chronic psychosocial distress was negatively correlated with serum levels of BDNF (Mitoma et al., 2008). Moreover, plasma BDNF reductions elicited by an acute stressor were prevented by pretreatment with the SSRI paroxetine (Tamaji et al., 2012). However, despite the considerable interest in BDNF as a potential contributor to the evolution of depression secondary to stressful events, it seems that this relationship is not always straightforward. In fact, serum increases of the neurotrophin have also been reported in response to an acute psychosocial stressor in humans (Meng et al., 2011). It is likely that the effects of stressors on BDNF in clinical populations may be influenced by several factors, including the stressor conditions, previous stressor experiences, and individual difference factors. In addition, these clinical data have, understandably, come from studies that evaluated BDNF changes in blood or from studies among individuals with particular polymorphisms of the BDNF gene (as will be discussed later). The peripheral index of BDNF has been related to central concentrations of the neurotrophin in rats (Angelucci et al., 2011; Klein et al., 2011), but it was also reported that BDNF in brain was unrelated to that evident in plasma (Kyeremanteng et al., 2012). Even if they were correlated, the plasma BDNF levels might not be informative with respect to the specific brain regions that were most responsible for the changes seen peripherally, a fact that is of particular concern as it has been suggested that the stressor effects on BDNF may be specific to particular brain region. This said, the data concerning peripheral BDNF in relation to stressor effects in humans have been inconsistent, and what these differences might mean with respect to subsequent neurogenesis, neuroplasticity, and depression, remain to be clarified.

Several animal studies have made it clear that depressive-like behaviors provoked by acute (e.g., restraint, social defeat) or chronic (e.g., chronic mild stress, prolonged immobilization) stressors were accompanied by decreased hippocampal neurogenesis (Tanti et al., 2012) as well as down-regulated BDNF expression in the hippocampus and the PFC (Smith et al., 1995; Ueyama et al., 1997; Pizarro et al., 2004). Contrary to a BDNF perspective of stressor-induced depression, however, BDNF elevations in the mPFC, amygdala, and substantia nigra have been reported after intermittent social defeat (Fanous et al., 2010), as well as in the neocortex and the nucleus accumbens after a chronic social stressor (Berton et al., 2006; Schulte-Herbrüggen et al., 2009). Once again, as in the clinical studies, the direction of BDNF changes elicited in animals appears to vary across stressor paradigms and as a function of the specific brain region examined.

It has been known for some time that different stressors can call upon diverse neural circuits, and may elicit very different behavioral outcomes (Anisman and Matheson, 2005). Thus, there has been some question as to which particular stressors should be applied in modeling specific psychopathology. A favorite that has been used in the case of depression is social defeat, as it represents a naturalistic stressor that engenders profound behavioral impairments as well as marked cytokine, growth factor, and neurochemical variations. In mice, acute social defeat down-regulated BDNF mRNA expression in the hippocampus, piriform cortex, and basolateral amygdala (Pizarro et al., 2004). Interestingly, latency to escape from the aggressor during a social defeat episode was negatively correlated with BDNF mRNA expression in the hippocampus (but not in amygdala), suggesting that an active response strategy during stressor exposure might be associated with higher hippocampal BDNF (Arendt et al., 2012). Likewise, when defeat was experienced chronically, depressive-like behaviors and hippocampal BDNF protein reductions were apparent only in those mice that displayed a passive profile during agonistic interactions (Gómez-Lázaro et al., 2011). In line with the view that elevated BDNF might promote active responding and better behavioral outcomes, genetic knockdown of BDNF in the ventral tegmentum enhanced vulnerability to the depressive-like effects of social defeat (Krishnan et al., 2007), whereas BDNF over-expression in the hippocampus was associated with resiliency in response to chronic mild stressor exposure (Taliaz et al., 2011). Thus, it is possible that intact or enhanced BDNF (existing prior to stressor exposure or elicited by the stressor), particularly in the hippocampus, might encourage the use of appropriate defensive strategies or perhaps more flexibility in adopting these strategies, thus promoting stressor resilience, and reducing the likelihood of depression emerging. In the presence of lower BDNF, the opposite outcomes might occur (e.g., inflexibility in adopting appropriate response methods), thus favoring stronger adverse stressor effects and greater vulnerability of developing depressive features.

It has usually been thought that uncontrollable stressors elicit more profound biological disturbances than do controllable stressors. Whereas both types of challenges elicited comparable reductions of BDNF mRNA expression in the hippocampus, in the PFC both escapable and inescapable shock increased BDNF expression, but the BDNF up-regulation was more pronounced among animals that had been exposed to the controllable stressor (Bland et al., 2007), thus suggesting that the BDNF changes elicited by shocks in the PFC might be associated with the degree of control/flexibility animals had over the stressor situation. Significantly, as well, BDNF elevations that occurred after psychosocial stressors varied as a function of the dominance status exhibited during agonistic encounters. Among submissive hamsters, BDNF was up-regulated in the amygdala, whereas in those hamsters that were dominant, BDNF elevations were apparent in the hippocampus (Taylor et al., 2011). Similarly, BDNF expression was elevated in the SVZ and hippocampus among aged dominant mice but not among their submissive counterparts (Fiore et al., 2003). Clearly, the BDNF variations elicited by stressful social interactions might be tied to inter-individual factors related to dominance or to the methods of dealing with or responding to stressors that require a certain control.

Summarizing briefly, the proposition that BDNF is altered in stress responses and depression has received substantial support. Indeed, it is even thought that the rapid antidepressant effects of the NMDA receptor antagonist ketamine might stem from its stimulating action on BDNF (Liu et al., 2012b). Yet, there are still data that don't align nicely with a straightforward version of the BDNF hypothesis of depression. Either there's less to this hypothesis than initially meets the eye, or, more probably, there are moderating factors that determine whether, and under what circumstances, BDNF might be aligned with depression. It is possible, for instance, that the effects of environmental (or experiential) events might be enhanced or made more cogent in the presence of unaltered or elevated BDNF, and as a result neuroplasticity would be particularly affected by these events. As well, experiencing a stressor might elicit a temporary effect on BDNF (e.g., to help coping with the stressful situation) that is likely to change with the passage of time and/or with further stressful experiences. Certainly, the influence of BDNF is likely to be dictated by the presence of still other endogenous substrates, including other growth and inflammatory factors and/or, as will be discussed in the next section, particular gene polymorphisms.

### BDNF: genetic influences on depression

In addition to being influenced by stressors, BDNF might also play a significant role in how individuals respond to stressors (behaviorally and physiologically) and might thus also contribute to whether these responses culminate in depression. In fact, it was suggested that the impact of stressful-life events on the development of growth factor impairments and depressive illnesses might be modulated by gene polymorphisms that influence neuroplasticity in response to environmental challenges (Belsky and Pluess, 2009). In this regard, child abuse and neglect among individuals with lifetime depression was associated with lower serum BDNF levels among carriers of the BDNF Val^66^Met polymorphism but not among those homozygous for the Val allele (Elzinga et al., 2011). Although there have been several comparable reports, it has also been shown that the presence of the Met allele might not necessarily lead to BDNF reductions that dispose individuals to depressive disorders. As a matter of fact, early adversity did not affect plasma BDNF levels in carriers of the Met allele nor in those with a mutation of the 5-HT transporter (5-HTT) in which a short (s) form of the 5-HTTLPR allele was present. However, among those individuals homozygous for both the BDNF Val and the 5-HTTLPR long (l) alleles, marked plasma BDNF reductions and increased susceptibility to depression were evident in association with early life adversity (Buchmann et al., 2012). Similarly, in adulthood, high depressive scores after chronic stressor experiences were more pronounced among individuals with the BDNF Val/Val genotype than in those carrying the Met allele (Jiang et al., 2012). Furthermore, among those homozygous for the 5-HTTLPR ss alleles, which are a risk factor for later depression provided that early life or adult stressors are encountered, the presence of the BDNF Met allele actually had a protective effect on mental health among those with a history of childhood abuse (Grabe et al., 2012).

Based on the findings regarding the relationship between low BDNF and depression, and the role of early and/or adult stressful events in promoting depressive symptoms, it had been suggested that gene polymorphisms should not be considered as a “vulnerability factor,” but should instead be viewed as a “plasticity factor” that may favor either positive or negative outcomes depending upon the individuals specific experiences (Belsky and Pluess, 2009; Belsky et al., 2009). Thus, as suggested in the case of 5-HTTLPR (Belsky et al., 2009), elevated BDNF may, “for better or for worse,” allow for particular experiences to mold neuronal processes, thereby favoring or buffering against psychological disturbances in response to further challenges. In effect, in the presence of the Val/Val alleles, negative experiences or an impoverished environment would lead to poor psychological well-being, whereas nurturing environments would lead to enhanced psychological well-being and resilience in the face of later stressors. In the presence of the Met allele, however, reduced plasticity (related to low BDNF activity) might itself favor the emergence of depression, but it would also limit the damage that could otherwise be inflicted by adverse early life experiences. It could also be argued from this perspective that the positive effects of a nurturing environment might, unfortunately, also have less of a positive effect in Met carriers.

### Epigenetic impact on BDNF variations in major depression

An obvious question that exists concerns how changes of BDNF levels and expression come about. From what has been said to this point, stressors can instill such variations and gene polymorphisms may influence whether and to what extent these changes will occur. However, to account for pathologies such as depression, PTSD, or addiction, it will be important to identify variables or processes that result in long-lasting changes of these processes. In this regard, epigenetic changes associated with adverse early life experiences as well as those that occur in adulthood have not only been documented with respect to glucocorticoid receptors (Francis et al., 1999; Weaver et al., 2004; Szyf et al., 2005; Champagne, 2010) or GABA receptor subunits (Poulter et al., 2008; Papadopoulos et al., 2011), but also in regard to BDNF. Specifically, increased DNA methylation of BDNF and altered prefrontal BDNF expression were reported in adult rats that had been stressed during the first week after birth, and these epigenetic BDNF alterations were transmitted to the next generation of rats (Roth et al., 2009; Roth and Sweatt, 2011). Likewise, strong stressors administered in adulthood diminished levels and transcriptional activity of BDNF and promoted altered DNA methylation and histone acetylation (Tsankova et al., 2006; Covington et al., 2009, 2011; Fuchikami et al., 2009, 2010). As expected, administration of a histone deacetylase (HDAC) inhibitor into the nucleus accumbens shell (Covington et al., 2009) or the hippocampus (Covington et al., 2011) attenuated the alterations of histone acetylation as well as depressive-like behaviors elicited by social defeat. Similarly, reduced hippocampal acetylation of the acetylcholinesterase gene elicited by a stressor was reversed by daily administration of an HDAC inhibitor (Sailaja et al., 2012). The use of HDAC inhibitors in the treatment of depressive illnesses thus seems promising, although the specific relations between these agents, BDNF variations, and depressive outcomes, remain to be fully clarified.

## Contribution of additional growth factors to depression: beyond BDNF

The case for BDNF involvement in depression has generally been impressive (Duman and Aghajanian, 2012) but as discussed, the available data do not fully conform to an accounting based solely on BDNF-mediated neurogenesis or neuroplasticity. Although their contribution to depression has not been assessed as extensively as that of BDNF, several reports have pointed to a role for additional growth factors in depressive disorders, including FGF-2, NGF, VEGF, and EPO (see Table 2), as well as macrophage migration inhibitory factor (MIF), which is considered as a pro-inflammatory cytokine that has neurogenic actions. These growth factors, to varying degrees, have been related to depressive disorders, although in the case of VEGF, MIF, and EPO it does not seem as if they systematically vary in depressed patients. Nevertheless, it appears that their administration can improve depressive symptoms, and in some case they might be a necessary condition for the effects of other antidepressants to be effective.

In the case of FGF-2, down-regulated expression of the protein and up-regulated expression of its receptor, FGFr1, were reported in post-mortem frontal cortex and hippocampus of patients who had been depressed (Evans et al., 2004; Gaughran et al., 2006). Limited FGF-2 down-regulation was apparent in those patients that had been treated with antidepressants (Evans et al., 2004), a finding also seen in mice in which antidepressants increased FGF-2 expression and immunoreactivity in hippocampus and PFC (Gómez-Pinilla et al., 2000; Maragnoli et al., 2004; Bachis et al., 2008; Elsayed et al., 2012). Furthermore, blockade of FGF-2 receptor signaling (Elsayed et al., 2012) or genetic knockout of FGF-2 (Jarosik et al., 2011) antagonized the behavioral effects of antidepressants in rodent models of depression, suggesting that this growth factor may be necessary for antidepressant effects to occur. A striking confirmation of FGF-2 involvement in mediating antidepressant outcomes is that prefrontal infusion (Elsayed et al., 2012) or intraventricular microinjections (Turner et al., 2008) of this growth factor in mice produced antidepressant effects and attenuated impairments of hippocampal neurogenesis elicited by olfactory bulbectomy (Jarosik et al., 2011) or a chronic unpredictable stressor (Elsayed et al., 2012).

The data regarding NGF involvement in depression have been more sparse than those involving BDNF and FGF-2. Nonetheless, reduced NGF levels in plasma were observed among drug-free depressed patients (Xiong et al., 2011a) and intranasal NGF administration in rats exerted antidepressant-like effects (Shi et al., 2010). Interestingly, however, enhanced plasma NGF levels were observed after acute and chronic emotional stressors in humans (Aloe et al., 1994; Hadjiconstantinou et al., 2001), thus how NGF variations play out in relation to stressor-elicited affective states is uncertain.

Unlike BDNF, NGF, and FGF-2, which were reduced in association with depression, plasma levels of VEGF were elevated (Lee and Kim, 2012) or unchanged (Ventriglia et al., 2009) in drug-free patients with an acute episode of major depression and VEGF levels were unrelated to depression scores. In fact, the only indication of a possible reduction of VEGF levels in relation to depression comes from reports showing that cerebrospinal fluid (CSF) VEGF levels were lower in medication-free suicide attempters compared to healthy controls and that low CSF VEGF levels were negatively correlated with depression severity (Isung et al., 2012a), and more pronounced among those individuals who later completed suicide (Isung et al., 2012b). As expected, however, concentrations of VEGF increased in remitted depressed patients that had been medicated (Takebayashi et al., 2010), but these elevations were limited in treatment resistant patients (Carvalho et al., 2012). Yet, it was also reported that VEGF levels were not altered during SSRI treatment despite clinical improvements (Ventriglia et al., 2009; Dome et al., 2012). Although a uniform picture regarding VEGF variations in depressed patients has yet to emerge, it seems that this growth factor may be required for an antidepressant effect to be realized, as several antidepressant treatments up-regulated hippocampal VEGF expression, and blockade of VEGF inhibited cell proliferation in the SGZ and greatly limited antidepressant outcomes (Warner-Schmidt and Duman, 2007).

Research concerning MIF in relation to depressive disorders has similarly led to inconsistent observations. Although circulating MIF levels and expression were elevated in depressed patients (Musil et al., 2011; Cattaneo et al., 2013), exogenous administration of this factor increased BDNF and FGF-2 expression *in vitro* and elicited antidepressant effects *in vivo* (Moon et al., 2012). Moreover, genetic deletion of MIF blocked the increased cell proliferation normally elicited by fluoxetine (Conboy et al., 2011), and limited the antidepressant effects and the increased hippocampal BDNF expression ordinarily elicited by exercise (Moon et al., 2012). It thus seems that although the findings concerning MIF in blood are not congruent with the potential involvement of this factor in depressive disorders, a better case exists for central involvement of MIF in depression. Nonetheless, even if MIF is not involved in the provocation of depressive symptoms, pharmacologically increasing its levels could still be a promising therapeutic strategy owing to its actions on other growth factors or neurotransmitters that influence the course of the illness.

Like MIF, there is reason to believe that EPO could serve in a therapeutic capacity in the treatment of depressive disorders. It is uncertain whether or not EPO in brain tissue is altered among depressed patients, although it was reported that this factor was elevated in the CFS (but not in serum) of depressed individuals and that chronic antidepressants reduced CSF EPO levels (Nakamura et al., 1998). Yet, EPO treatment in rats produced antidepressant effects (Girgenti et al., 2009) and in clinical trials the administration of EPO alleviated depressive symptoms (Miskowiak et al., 2012).

Most of the research on the implication of growth factors in depressive illnesses has concerned BDNF, although increasing efforts are now being devoted to examine the role of additional growth factors in depression and/or their contribution to the positive outcomes of antidepressants. Whereas each growth factor might exert particular actions, it is likely that a combination of growth factor variations (rather than impairment of a unique marker) is involved in the pathogenesis of depressive symptoms as well as in the clinical outcomes of antidepressant treatments. Currently, however, the specific contributions of each growth factor to depression and their potential interactions in the course of the illness remain to be clarified.

## Effects of pro-inflammatory cytokines on neurogenesis and BDNF: relevance to depression

It will be recalled that increased activity of pro-inflammatory cytokines and impaired neurogenesis (including reduced BDNF functioning) are apparent in drug-free depressed patients and that clinical improvements resulting from antidepressant treatments have been associated with normalization of particular cytokines and growth factors. The question remains as to whether these systems act in parallel or interactively in relation to the promotion and/or the amelioration of depressive illnesses. It has been suggested that increased activity of pro-inflammatory cytokines might alter growth factors, and thus neurogenesis and plasticity, culminating in depression. It is, indeed, known that inflammatory responses elicited by immune challenges and/or stressors may damage microglia and neurons, although it is still uncertain which mechanisms are responsible for such outcomes.

Treatment with the bacterial endotoxin LPS not only has peripheral effects on immune functioning, but also instigates several changes within the brain. Of particular significance to the present discussion is the finding that acute LPS treatment diminished the number of new neurons generated (Ormerod et al., 2013), impaired the proliferation of hippocampal precursor cells (Fujioka and Akema, 2010), and transiently reduced BDNF and NGF levels in the cortex and hippocampus (Guan and Fang, 2006; Kranjac et al., 2012). Impairments of hippocampal neurogenesis induced by LPS were prevented by antidepressants (Peng et al., 2012) and, importantly, were completely blocked by the non-steroidal anti-inflammatory agent indomethacin, confirming the contribution of inflammatory processes to impairments of neurogenesis (Monje et al., 2003). In contrast to acute microglial activation by LPS, persistently activated microglia did not elicit disturbances of neurogenesis, probably owing to the limited pro-inflammatory cytokine elevations in chronically stimulated cells (Cacci et al., 2008). Thus, it is possible that the degree of microglia activation resulting from an immunogenic (or stressor) challenge might be fundamental in determining the extent of disturbed neurogenesis. In this regard, it has long been considered that moderate and transient cytokine elevations might be neuroprotective, whereas relatively high and persistent levels might be neurodestructive.

In addition to the non-specific microglia activation elicited by LPS, administration of particular pro-inflammatory cytokines may directly affect neurogenesis, and it seems that IL-1β might be especially potent in this regard. *In vitro* application of IL-1β to neural stem cells reduced their differentiation (Kuzumaki et al., 2010), decreased their maturation, and promoted proliferation of undifferentiated cells (Zunszain et al., 2012). Conversely, reduction of IL-1β expression with the toxin, Cytotoxic Necrotizing Factor 1 promoted neuritogenesis and synaptogenesis (Malchiodi-Albedi et al., 2012). Likewise, pre-treatment with the IL-1β antagonist IL-1ra reversed the impaired neurogenesis elicited by *in vitro* IL-1β or by stressors (Koo and Duman, 2008) as well as the down-regulated hippocampal BDNF and cognitive impairments elicited by social isolation in mice (Barrientos et al., 2003).

Complicating the view that increased cytokine activity impairs neurogenesis, it seems that under certain circumstances, acute administration of pro-inflammatory cytokines may have neuroprotective effects. In fact, like IL-1β, *in vitro* administration of IL-6 or TNF-α (Monje et al., 2003; Cacci et al., 2005) or chronic secretion of IL-6 by microglia in adult transgenic mice (Vallières et al., 2002) reduced hippocampal neurogenesis and cell survival. However, *in vitro* application of TNF-α was also shown to increase proliferation and differentiation of neural stem cells (Widera et al., 2006; Bernardino et al., 2008), and to up-regulate exon-IV-bdnf mRNA as well as BDNF protein in primary astrocytes, probably through its actions on NF-κ B (Saha et al., 2006). Moreover, secretion of IL-6 from LPS-activated astrocytes promoted proliferation (Wang et al., 2011b), whereas in adult mice lacking IL-6, proliferation and survival of hippocampal progenitors were compromised (Bowen et al., 2011). Interestingly, the neuroprotective versus neurotoxic effects of TNF-α seemed to depend on the receptor subtype activated by the cytokine, in that stimulation of the TNFR1 receptor inhibited neurogenesis, whereas that of the TNFR2 increased proliferation and survival of neural stem cells (Iosif et al., 2006). Unlike TNF-α, the differential effects exerted by IL-6 on neurogenesis appeared to vary as a function of the duration of exposure to the cytokine and other experimental conditions (e.g., concentration or amount of IL-6) (Molina-Holgado and Molina-Holgado, 2010), consistent with the observation that moderate versus high microglia activation might differential influence neurogenesis, although at present the data regarding the dual effects of IL-6 are still sparse, thus precluding definitive conclusions.

In keeping with the view that increased pro-inflammatory cytokine activity might promote depression through their effects on growth factors, serum BDNF reductions during the course of IFN-α treatment were inversely correlated with depressive scores (Kenis et al., 2011), although it has also been found that depression severity during IFN-α immunotherapy was associated with lower BDNF levels prior to treatment, but not with the BDNF reductions that occurred during treatment (Lotrich et al., 2013). Moreover, basal serum levels of sIL-2r and of IL-1ra predicted BDNF reductions during IFN-α therapy, but the two systems were independently associated with the development of depression during treatment (Kenis et al., 2011). Cytokine-induced impairments of neurogenesis and growth factors have also been reported to affect neurochemical processes that had been directly implicated in depression. Application of IL-1β to hippocampal stem cells blocked their differentiation into serotonergic neurons and decreased 5-HT concentrations normally released in differentiated cells, an effect that was prevented by IL-1ra (Zhang et al., 2013). While decreasing neurogenesis of hippocampal progenitors, *in vitro* application of IL-1β also up-regulated IDO as well as enzymes involved in the neurotoxic effects associated with the kynurenine pathway, ultimately limiting tryptophan availability and promoting neurotoxicity (Zunszain et al., 2012). At this time there is insufficient information available to firmly conclude that pro-inflammatory cytokines promote depression through their actions on BDNF or other growth factors. To be sure, it does appear that pro-inflammatory cytokines, like stressors, may influence BDNF, and it likewise seems that under certain conditions BDNF may be related to depression. However, the linkage between these factors, and the conditions under which this sequential series of changes occurs, has yet to be fully defined.

## Pro-inflammatory cytokine and BDNF interactions following stroke: relevance to post-stroke depression

It would be of considerable interest to evaluate the temporal variations of pro-inflammatory cytokines and growth factors and their relationships among depressed patients in the absence of other comorbid conditions or in animal models of depression. However, the data concerning the interplay between these factors has largely come from studies assessing processes that occur following cerebral damage stemming from traumatic brain injury (TBI) or stroke, especially those variations that occur in the brain. In this regard, it has been suggested that pro-inflammatory cytokines released after brain trauma or injury could initially promote neural generation owing to the growth factor elevations and/or the neural stem cells that are recruited to the site of damage in response to the cytokine elevations or, under specific circumstances, impair neurogenesis and neuroplasticity and compromise clinical and functional recovery (Nakatomi et al., 2002). The beneficial versus detrimental effects of pro-inflammatory cytokines on neurogenesis after brain damage are particularly relevant as a substantial proportion of individuals that suffer from TBI or stroke will experience depressive symptoms during the course of recovery (Whyte and Mulsant, 2002) and the presence of depression may actually predict poor prognosis for functional recovery and global functioning (Bilge et al., 2008).

In this regard, elevations of circulating pro-inflammatory cytokines following central damage in the form of stroke were associated with later emergence of depression (Yang et al., 2010), poor prognostic outcomes (Vila et al., 2000; Whiteley et al., 2009), and predicted mortality at 12 months (Shenhar-Tsarfaty et al., 2010). In particular, serum IL-18 levels, but not that of IL-6 or TNF-α, measured 1 and 7 days after stroke were higher in patients that later developed depression (Yang et al., 2010). Further, serum IL-1β, IL-1ra, and IL-9 levels measured within 72 h after stroke predicted fatigue symptoms (Ormstad et al., 2011), but not depressive symptoms (Ormstad et al., 2012). Beyond the possible involvement of pro-inflammatory cytokines, lower concentrations of the anti-inflammatory cytokine IL-10 measured shortly after an ischemic stroke were associated with early neurological deterioration (Vila et al., 2003). Likewise, risk of depression following stroke was enhanced among individuals who carried alleles associated with reduced anti-inflammatory cytokine functioning (IL-4 and IL-10 gene polymorphisms), although no such association was found with respect to pro-inflammatory cytokine polymorphisms (Kim et al., 2012b).

It is interesting that those peripheral cytokines that predict emergence of depression after a stroke (e.g., IL-18) differ from the key cytokines typically implicated in depressive illnesses that occur in other circumstances (e.g., IL-6, TNF-α, or IL-1β). Nevertheless, a prospective examination of cytokine changes during post-stroke recovery revealed that among those patients that expressed depressive symptoms one year after stroke, serum concentrations of IL-6, IL-10, TNF-α, and IFN-γ were concomitantly increased, supporting the supposition that these key cytokines might contribute to, or be an index of, post-stroke depressive symptoms (Su et al., 2012). Although in this study serum IL-1β levels were too low to be detected (Su et al., 2012), a role for IL-1β in post-stroke depressive symptoms has been confirmed in animal models in which the anhedonia elicited by middle cerebral artery occlusion was attenuated by intracerebrovascular injection of IL-1ra (Craft and DeVries, 2006).

As in the case of major depression, the mood symptoms that follow stroke have been related to impairments of BDNF. In this regard, a meta-analysis indicated that lower serum BDNF levels (measured within a mean of 2 months after stroke) were associated with the presence of post-stroke depression (Noonan et al., 2012). Moreover, the 5-HTTLPR ss and the BDNF Met–Met genotypes, both of which had been implicated in major depressive disorder, were also associated with the presence of depression two weeks following stroke (Kim et al., 2012a) as well as with poor prognostic outcomes and worsened physical disability and cognitive functions two weeks and one year after stroke (Kim et al., 2012c). Paralleling these findings, in animal models, locomotor deficits and reduced angiogenesis elicited by ischemic stroke were accentuated in those animals with the Met–Met polymorphism (Qin et al., 2011).

Although BDNF reductions in stroke patients and animal stroke models were correlated with functional impairments and depressive symptoms, very shortly after cerebral ischemia, BDNF expression in the contralateral and ipsilateral damaged cortex transiently increased, although in the hippocampus BDNF elevations were delayed (Madinier et al., 2013). This BDNF rise might have reflected a transient adaptive or compensatory change as the neurotrophin subsequently declined, especially in cortical areas (Madinier et al., 2013). In fact, shortly after ischemic stroke, endogenous neural stem cells proliferated, migrated into the damaged area, and generated new neurons (Nakatomi et al., 2002). It thus appears that the release of both pro-inflammatory cytokines and BDNF may be transiently enhanced after stroke, possibly to protect damaged neuronal tissue (Nakatomi et al., 2002; Broughton et al., 2012; Madinier et al., 2013). As in the case of peripheral inflammation elicited by an endotoxin (Cacci et al., 2008), the neuroprotective versus neurotoxic/neurodegenerative effects of pro-inflammatory cytokine elevations following brain damage might depend upon the degree and duration of microglia activation and the ensuing impact on neurogenesis and related factors, such as BDNF. Interestingly, in rats, administration of BDNF shortly after ischemic stroke increased brain levels of the neurotrophin and prevented neuronal loss and motor deficits that would have otherwise occurred, an effect that was mediated by the up-regulated IL-10 and down-regulated TNF-α expression elicited by the neurotrophin (Jiang et al., 2010, 2011). As described earlier, it thus seems likely that excessive levels of cytokines and/or reductions of BDNF, particularly if they persist for extended periods, as well as the balance between these factors, might be keyed in determining whether positive or negative outcomes emerge after stroke.

As alluded to earlier, MIF and EPO might be related to depressive disorders, although the available data are admittedly limited. It also seems that these factors may be related to functional outcomes stemming from stroke or TBI, especially as they are inducible by hypoxia (Chin et al., 2000; Wang et al., 2009). In fact, elevated serum and brain MIF during the first days following stroke were positively correlated with the severity of brain damage both in patients (Wang et al., 2009) and in a rat stroke model (Inácio et al., 2011a,b). Moreover, in mice with a genetic deletion of MIF (Inácio et al., 2011b) or in which MIF was diminished following housing in an enriched environment (Inácio et al., 2011c), smaller infarct size and a better functional outcome were apparent after transient focal ischemia, pointing to a role for MIF in neuronal death and in functional deficits elicited by stroke. Unexpectedly, it seems that MIF variations after stroke might occur independently of changes of other pro-inflammatory cytokines as genetic deletion of MIF did not affect ipsilateral IL-1β up-regulation (in the infarct core and peri-infarct area) or serum and brain TNF-α elevations that also occur after stroke (Inácio et al., 2011a,b). Unfortunately, as far as we know, data have not been reported concerning MIF in relation to post-stroke depression. As MIF has also been implicated in the up-regulation of BDNF and FGF-2 *in vitro* and in the antidepressant effects of different treatments *in vivo* (Moon et al., 2012), the data regarding neurotoxic effects of MIF are incongruous and certainly necessitate further investigation. If nothing else, it is necessary to track the variations of MIF levels over time following stroke and determine to what extent these might be related to changes of other cytokines or growth factors and ultimately, to particular depressive and other cognitive outcomes. As indicated earlier, the levels of cytokines and the damage or beneficial effects that occur, might vary in a dose-dependent bimodal fashion, and could potentially vary over time following the brain insult.

In contrast to MIF, EPO has been found to limit neuronal damage and contribute to functional recovery. In fact, both EPO and its receptor are expressed in various regions of the brain and their expression increased during and after ischemia (Sirén et al., 2001). Furthermore, EPO administration after ischemic stroke or TBI increased BDNF and VEGF expression and improved functional recovery (Wang et al., 2004; Xiong et al., 2011b). Such effects were likely not attributable to the hematopoietic effects of EPO, as treatment with EPO or with its carbamylated form (C-EPO), which does not influence erythropoiesis (production of red blood cells), also increased neurogenesis in the dentate gyrus and limited cell loss as well as spatial learning and sensorimotor deficits elicited by TBI (Mahmood et al., 2007; Xiong et al., 2011b). Interestingly, inhibition of VEGF receptor 2 abolished the neuroprotective effects of EPO after TBI (Xiong et al., 2011b), supporting a role for this growth factor in the functional improvements observed.

In addition to its enhancing actions on growth factors (Wang et al., 2004; Xiong et al., 2011b), it was suggested that increased EPO expression following brain damage could reduce local production of pro-inflammatory markers, possibly limiting further damage to surrounding tissues and thus preventing subsequent functional deterioration (Alnaeeli et al., 2012). Up-regulated serum and brain pro-inflammatory cytokines elicited by TBI or stroke were attenuated by treatment with rhEPO (Chen et al., 2007; Bian et al., 2010; Chau et al., 2011), although EPO administration enhanced BDNF up-regulation elicited by cerebral ischemia, but did not prevent inflammatory gene up-regulation (e.g., IL-1β, IL-6, TNF-α) (Mengozzi et al., 2012). As EPO has been implicated as a promising antidepressant treatment (Nakamura et al., 1998; Girgenti et al., 2009; Miskowiak et al., 2012), it appears important to assess its potential effects on both pro-inflammatory cytokines and growth factors. This said, several studies have been conducted concerning the safety and efficacy of EPO administered shortly after stroke. Intravenous high-dose rhEPO was well-tolerated among patient aged 80 and over that experienced acute ischemic stroke and was associated with improved clinical outcomes measured one month later (Ehrenreich et al., 2002). However, in other studies, EPO had little benefit, and in patients with cancer or chronic kidney disease, thromboembolic complications, and/or mortality risks were associated with EPO treatment (Ehrenreich et al., 2009). Thus, there may be some limitations as to when EPO can be used, and additional analyses would be necessary to determine other conditions where its use would be contraindicated.

## Summary and conclusions

**Figure 1:**
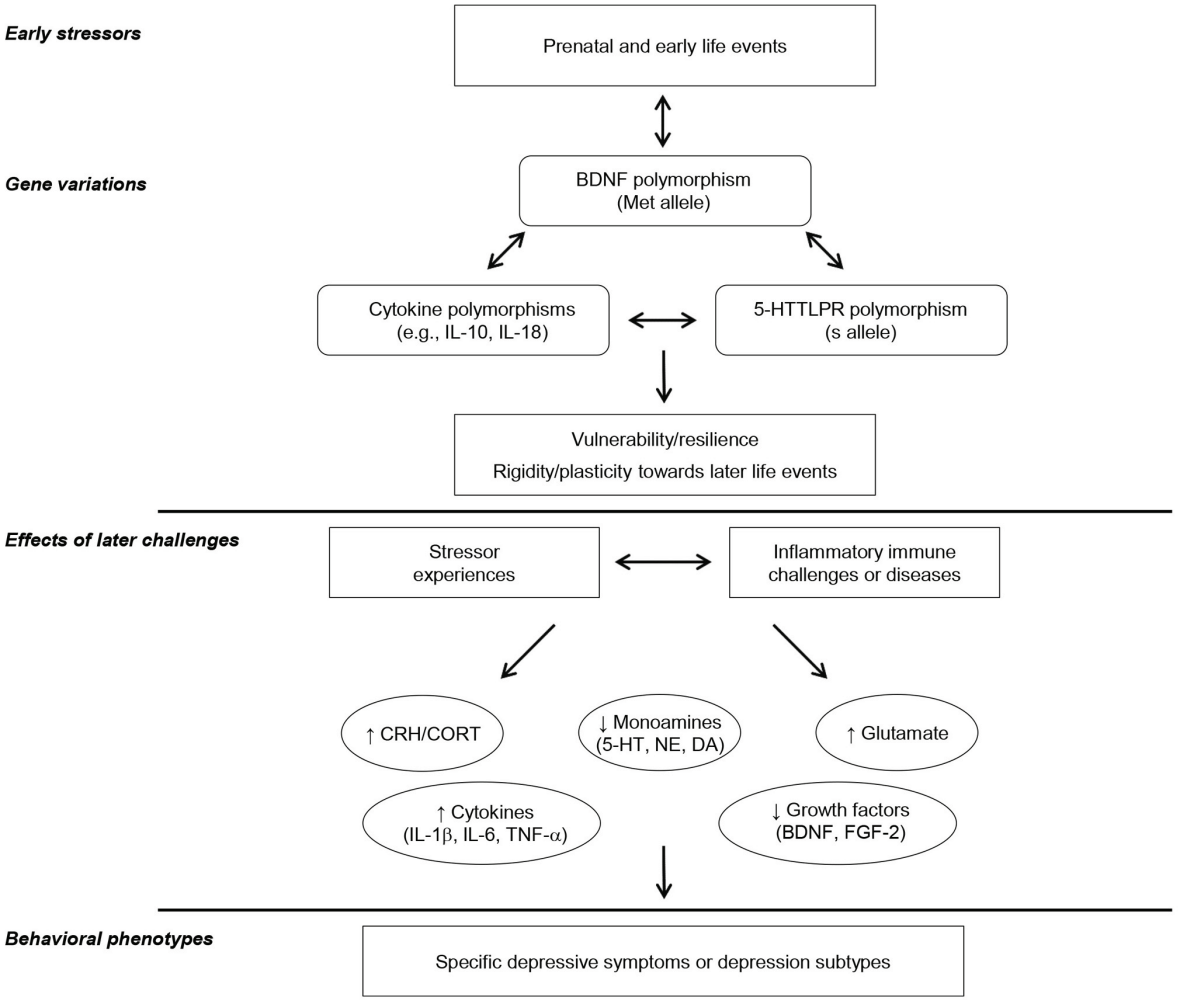
**This provides a schematic representation of the potential interactive influence of prenatal, childhood, and/or adulthood events and particular genetic variations in affecting inflammatory and growth factors, ultimately leading to specific depressive illnesses.** It is suggested that particular gene polymorphism, including that of the growth factor BDNF, may influence vulnerability versus resilience to the emergence of depression, and that this may be moderated by the presence of adverse childhood experiences. In this regard, gene polymorphisms are not seen as necessarily being related to positive or negative outcomes, but instead presence of adequate factors might permit the environment or particular experiences to mold subsequent illness vulnerability or resilience. Depending on the interactions between gene polymorphisms and the presence of early adverse experiences, subsequent exposure to stressful events or inflammatory immune challenges (e.g., chronic inflammatory diseases, post-stroke inflammation) would lead to sensitization of processes that elicit distinctive variations of brain monoamines, glutamate, growth factors, and/or pro-inflammatory cytokines and influence how they interact in promoting specific depressive phenotypes/subtypes.

The perspective that impairments of growth factor functioning, such as BDNF and others within the different families of growth factors, might contribute to major depression has gained increasing attention. However, it also seems that these growth factors, considered individually, might not fully account for the development of all of the symptoms comprising depressive illnesses. In fact, as indicated earlier, inflammatory processes might play a fundamental role in depression that arise in relation to stressful events, as well as depression that occurs in the course of chronic inflammatory diseases (e.g., chronic hepatitis C) or of post-stroke or TBI recovery. It is of particular interest that growth factors, especially BDNF, and pro-inflammatory cytokines might have interactive effects on neurogenesis, depending on cytokine concentrations (and duration of their activation), although it is uncertain whether they act together or independently in the provocation or exacerbation of depressive illnesses and/or in contributing to the clinical outcomes of antidepressants. As depicted in Figure 1, prenatal and early adverse experiences as well as the presence of particular gene polymorphisms, including that of BDNF, may interact to promote resilience and/or plasticity in the face of later stressor experiences, or inflammatory immune challenges or diseases. These variations, in turn, might promote brain changes of corticotropin-releasing hormone (CRH), monoamines, glutamate, pro-inflammatory cytokines, and growth factors any of which could contribute to particular features of depressive illnesses.

The moderate efficacy of SSRIs and SNRIs in the treatment of depression invigorated the search for the processes associated with depressive disorders as well as the hunt for biomarkers that would foretell the occurrence of depression and predict the efficacy of antidepressant strategies. The perspective that inflammatory and growth factors might serve in these capacities has received considerable support, although it appears likely that some instances of depression might not be related to them entirely. For example, as indicated earlier, it seems that those individuals with high inflammatory cytokine levels might be less responsive to SSRIs and SNRIs. Thus, there is the possibility that anti-inflammatory agents could be useful as an adjunctive treatment in attenuating depressive symptoms in these patients, although their efficacy in this regard has been variable.

It seems that medication-free depressed patients may exhibit a unique profile in which activation of particular pro-inflammatory cytokines (especially that of IL-6) and reductions of growth factors (especially that of BDNF) are inversely related to the severity of depression. Yet, there are troubling findings that indicate that this supposition is too simplistic. It is possible that cytokine and/or BDNF contribute to particular symptoms of depression, or particular depressive subtypes. Despite the call for an endophenotypic approach to studying mental illnesses, few studies have focused on the link between specific inflammatory or growth factors and particular characteristics of depressive disorders. Ultimately, individualized treatment approaches might be most suitable in dealing with disorders that involve multiple, but varying symptoms and that seem biologically heterogeneous. At this time there is ample reason to suggest that particular cytokines (e.g., IL-6) and growth factors (e.g., BDNF) might serve as biomarkers in predicting who would be most prone to depression under various challenge conditions, and which treatment strategies would be most efficacious.

### Conflict of interest statement

The authors declare that the research was conducted in the absence of any commercial or financial relationships that could be construed as a potential conflict of interest.
